# Structural Significance of Hydrophobic and Hydrogen Bonding Interaction for Nanoscale Hybridization of Antiseptic Miramistin Molecules with Molybdenum Disulfide Monolayers

**DOI:** 10.3390/molecules28041702

**Published:** 2023-02-10

**Authors:** Alexander S. Goloveshkin, Natalia D. Lenenko, Alexander V. Naumkin, Alexandre S. Golub

**Affiliations:** A.N. Nesmeyanov Institute of Organoelement Compounds, Russian Academy of Sciences, Vavilova St. 28, 119934 Moscow, Russia

**Keywords:** organic–inorganic hybrids, molybdenum disulfide, layered compounds, quaternary ammonium surfactants, noncovalent interaction

## Abstract

This paper reports an easy route to immobilize the antiseptic drug miramistin (MR) molecules between the sheets of molybdenum disulfide, known for excellent photothermal properties. Two hybrid layered compounds (LCs) with regularly alternating monolayers of MR and MoS_2_, differing in thickness of organic layer are prepared and studied by powder X-ray diffraction (PXRD), X-ray photoelectron spectroscopy (XPS), density functional theory (DFT) calculations and quantum theory of atoms in molecules (QTAIM) topological analysis. The obtained structural models elucidate the noncovalent interaction network of MR molecules confined in the two-dimensional spacing surrounded by sulfide sheets. It emerged that the characteristic folded geometry of MR molecule previously evidenced for pure miramistin is preserved in the hybrid structures. Quantification of the energetics of bonding interactions unveils that the most important contribution to structure stabilization of both compounds is provided by the weak but numerous CH…S bonding contacts. They are accompanied by the intra- and inter-molecular interactions within the MR layers, with dominating bonding effect of intermolecular hydrophobic interaction. The results obtained in the models provide a comprehensive understanding of the driving forces controlling the assembly of MR and MoS_2_ and may lead towards the development of novel promising MoS_2_-based photothermal therapeutic agents.

## 1. Introduction

Molybdenum disulfide is a famous 2D van der Waals solid, which is considered as one of the most promising post-graphene materials owing to its outstanding properties. These properties are highly demanded for various applications such as advanced field effect transistors [1,2], electrocatalysts [3,4,5], biosensors [6] and energy storage materials [7,8]. Yet another application of MoS_2_ has attracted tremendous attention during the last few years, which is based on its excellent photothermal activity. The nanodispersed particles of MoS_2_ were found to effectively transform the absorbed near infrared (NIR) laser radiation to heat. This feature, coupled with easy penetration of NIR radiation through human skin and tissue, initiated construction of novel MoS_2_-based agents for photothermal therapy, including antitumor, antimicrobial and fungicidal treatments. By design, the MoS_2_ particles are meant to induce a local hyperthermic effect upon irradiation and thus help to destroy undesirable cells or pathogens. In most such agents, MoS_2_ is combined with the chemotherapeutic organic compounds intended for corresponding treatments to achieve a synergetic effect. For instance, the MoS_2_ assemblies with a dinitroso-derivative of para-phenylenediamine [9] or polyethylene glycole [10] showed high efficacy in wound therapy. In this respect, the design of new MoS_2_-organic compounds with drug molecules and profound structural insight into the bonding interactions occurring in such systems are highly topical research fields.

During recent years, a number of MoS_2_ layered compounds with organic species have been prepared by the soft chemical exfoliation-restacking method [4,11], hydrothermal synthesis [5,12] or surface functionalization [13,14]. Structural modeling and density functional theory (DFT) calculations performed for some such systems have evidenced a strong noncovalent bonding between the MoS_2_ sheets and organic molecules as, for example, alkyl(aryl)amines [11,12], phenanthroline [4], phthalocyanine [15] and guanidine derivatives [16]. However, the structural and bonding peculiarities of the MoS_2_ associates with drug molecules remain underexplored with some rare exceptions, such as the recently studied layered compound of MoS_2_ with protein kinase inhibitor imatinib [17]. The reason of impediment to the progress in knowledge-gathering in this field, and therefore prediction of novel effective photothermal agents, is a deficiency of reliable atomic-level structural models of MoS_2_-based hybrid compounds, containing complicated drug molecules. One of the most valid methods to obtain necessary experimental structural information for building up such models is the incorporation of corresponding organic species in the heterolayered structures with alternating MoS_2_ sheets and organic layers. Determination of the structural parameters of such compounds can help to model the packing of an organic monolayer adjoining to the sulfide sheet and to envisage the interatomic contacts at MoS_2_–drug layer interface [18].

**Figure 1:**
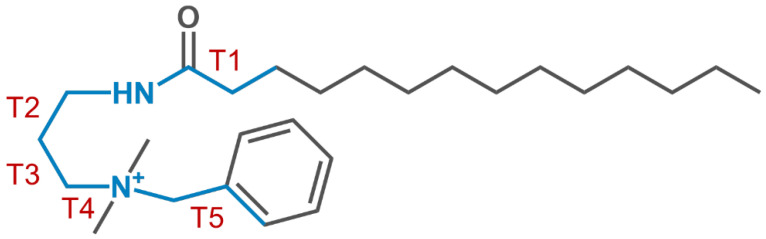
Chemical structure of miramistin with notation of torsion angles characterizing its stable bent conformation.

Quaternary ammonium benzalkonium-type surfactants containing a cationic ammonium head group with the benzyl- and long-chain aliphatic substituents are well known antiseptics [19]. The hydrophobic interaction of their aliphatic chains with the lipid membranes of microbial or fungi cells leads to the membrane softening and greatly enhances their permeability, finely destroying the pathogens. Miramistin is a broad-spectrum antiseptic of the above-mentioned type, the active moiety of which is the benzyldimethyl{3-[(1-oxotetradecyl)amino]propyl}ammonium [20], medicated as a solution of its chloride salt [20,21,22]. It was proven to be active against the various microbial and fungi infections [22,23,24] and is often considered as one of the benchmark drugs for corresponding chemotherapies [25,26]. The distinctive feature of MR molecular skeleton is the presence of amidopropyl linker, which is capable of playing the role of a hinge between the ammonium head and aliphatic tail (Figure 1). The conformation preference of this molecule and peculiarities of the packing geometry of its hydrophilic and hydrophobic parts have recently been determined by single-crystal X-ray diffraction [27]. Considering the potential usage of MR–MoS_2_ hybrid composites as combined photothermal and antiseptic materials, it was of utmost interest to study the mutual affinity of the components, in particular, to reveal the conditions for filling the MoS_2_ interlayer gaps with MR molecules, which have never been reported before.

With the aim to provide a scientific basis for involvement of Miramistin and related compounds in design of MoS_2_-based photothermal materials, we elucidated the capability of MR molecules towards self-assembly into two-dimensional nanostructures with MoS_2_ sheets produced by liquid-phase exfoliation of molybdenum disulfide crystals. In order to determine the structure of the resultant organic–inorganic architectures and to evaluate the significance of their intermolecular interactions, the powder X-ray diffraction (PXRD), X-ray photoelectron spectroscopy (XPS), density functional theory (DFT) calculations and quantum theory of atoms in molecules (QTAIM) analysis were applied.

## 2. Results

### 2.1. Assembly of Hetero-Layered Compounds

**Figure 2:**
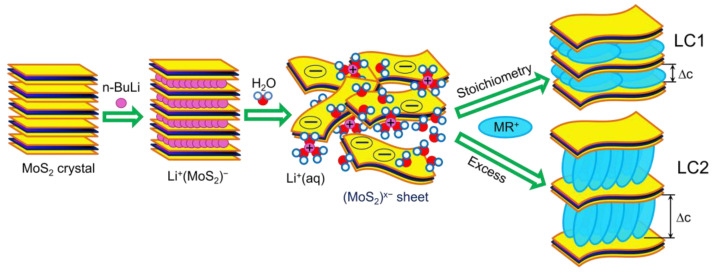
Schematics of assembly reaction of LCs.

**Figure 3:**
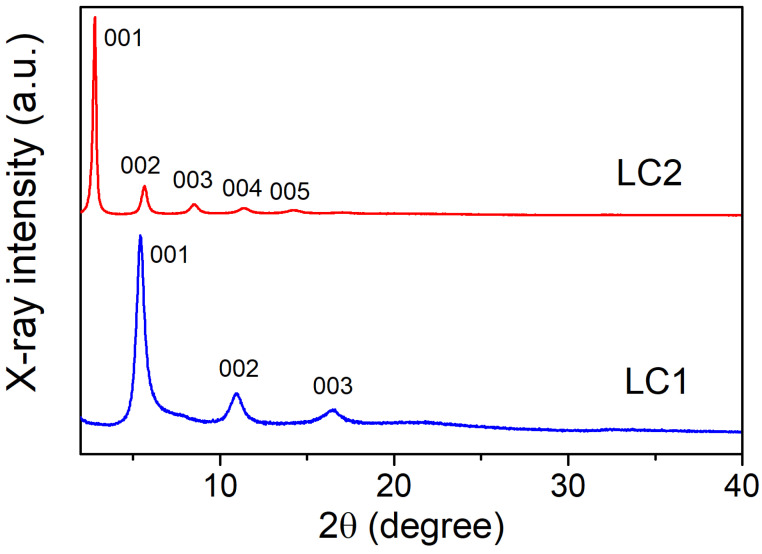
PXRD patterns of LCs with indication of 00*l* reflections.

For assembly of the hybrid MR–MoS_2_ structures, the interaction of MR salt with single-layer dispersions of MoS_2_ was used as this method was reported to be effective for obtainment of layered compounds (LCs) of MoS_2_ with some alkylammonium cations [28,29,30]. The schematics of the preparation process of LCs is shown in Figure 2. The exfoliation of the parent MoS_2_ crystals was achieved by their intercalation with Li followed by hydration, which produces negatively charged (MoS_2_)^x−^ sheets according to Equation (1). The MR cations were then allowed to react with the anionic sulfide sheets (Equation (2)) under different molar ratios. Similar to the early studied reactions of MoS_2_ single-layer dispersions with organic salts [28,30], the mixing with MR salt induced the flocculation of dispersions. The analysis of the formed precipitates has revealed that there are two stable phases are formed in this reaction. According to the PXRD data, both phases have layered structure, exhibiting by the family of intense 00*l* reflections, the positions of which determine the periodicity perpendicular to the MoS_2_ layer plan, i.e., the interlayer distance (*c*) (Figure 3). The first structure (referred to as LC1) has the composition (MR)_0.14_MoS_2_ and *c*-value of 16 Å. It can be prepared in monophasic form if the components are taken in stoichiometric amount, i.e., with the MR/MoS_2_ molar ratio (x) in reaction mixture equal to 0.14. Upon lesser or larger amount of MR in solution, a simultaneous formation of the non-intercalated MoS_2_ or phase with greater interlayer distance occurs, respectively. The latter phase (LC2) is isolatable as a stable (MR)_0.27_MoS_2_ compound with c = 31 Å upon excess of MR in solution. Comparison of the interlayer distance of the obtained LCs with that of the pristine non-intercalated MoS_2_ (6.15 Å), give the thicknesses of the embedded organic layers for these compounds (Δc), amounting to ~9.9 Å (LC1) and ~24.9 Å (LC2).

H_2_O           Li^+^(MoS_2_)^−^ ----------> [Li^+^ + (MoS_2_)^x−^ + (1 − x) OH^−^]*_aq_* (1)

(MoS_2_)^x−^ + x MR^+^ ----------> (MR)_x_MoS_2_ (2)

### 2.2. XPS Analysis

**Figure 4:**
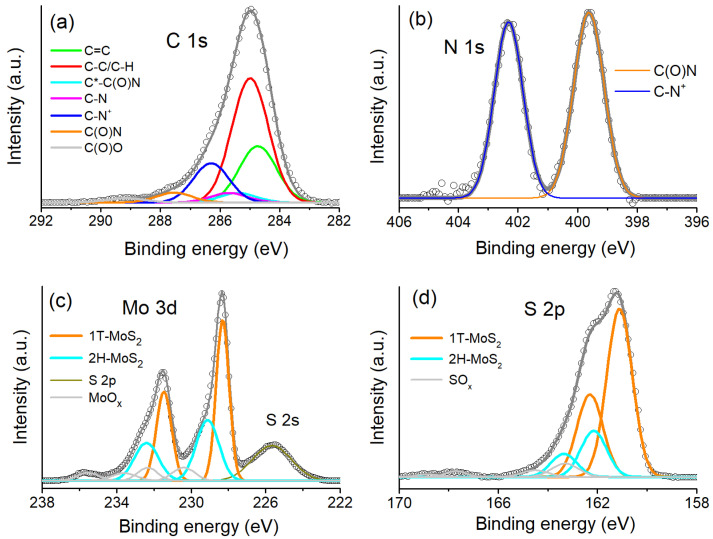
XPS spectra of LC1: C 1s (**a**), N 1s (**b**), Mo 3d (**c**) and S 2p (**d**).

XPS analysis of the prepared LCs has been performed in order to confirm their hybrid composite structure and determine the type of sulfur coordination environment of the Mo atoms in sulfide layers (octahedral or trigonal prismatic). The C 1s, N 1s, Mo 3d and S 2p spectra of LC1 are shown in Figure 3 and Figure S1; the fitting parameters are presented in Tables S1 and S2. The C 1s spectra were fitted with several Gaussian components in accordance with the chemical structure, using the reference data on chemical shifts [31]. The peaks at ~284.7, 285.0, 285.4, 285.7, ~286.2, ~287.6 and 289.2 eV are thus attributed to aromatic carbons, aliphatic carbons, secondary shift in C*–C(O)N and C*–C(O)O groups, C–N bonds, C–N^+^, C(O)N, C(O)O groups, present in MR molecules and in minor oxidized carbonaceous CO(O) impurity. Accordingly, the N 1s spectra fitted with two peaks at ~399.6 eV and ~400.3 eV reveals, respectively, the contributions of C(O)N and C–N^+^ groups of MR molecule. The intensity ratios of the spectral components in the C 1s and N 1s spectrum of LC1 (Figure 4a,b) agree well with those expected for MR molecule. Similar components were detected in the C 1s and N 1s spectra of LC2, with some deviations from LC1 in component ratios (Figure S1, Tables S1–S3). These deviations can be explained by the much higher screening effect for electron emission in LC2 caused by doubled charge density at MoS_2_-MR interface. Additionally, the difference in orientation of MR molecules in LC1 and LC2 discussed below produces different depth of occurrence of MR groups in these LCs relative to the specimen surface.

The Mo 3d spectra of LCs were fitted with four 3d_3/2_–3d_5/2_ spin-orbit doublets (Figure 4c and Figure S1c). The Mo 3d_5/2_ peaks located at ~228.3, 229.1, 222.3 and 230.4 eV are identified as 1T-MoS_2_ modification (octahedral Mo polyhedron), 2H-MoS_2_ (trigonal prismatic polyhedron), and impurity MoS_x_O_y_ and MoO_z_ states that is in accordance with previous studies [4,5,32]. The S 2p spectra fitted with four S 2p_3/2_–S 2p_1/2_ doublets are shown in Figure 4d and Figure S1d along with indication of the assigned components. Both the Mo 3d and S 2p regions thus clearly show the domination of octahedral 1T form in MoS_2_ layers of present LCs. Notice that the Mo/S ratios determined by the relative intensities of Mo 3d and S 2p XPS spectra of LCs do not change upon synthesis conditions and remain to be close to 2.0, similar to the initial crystalline stoichiometric MoS_2_.

### 2.3. Structural Modeling of LCs

In starting models of hybrid LCs, the layers of MoS_2_ had the 1T geometry with octahedral Mo-S coordination and zigzag chains in Mo sublattice in accordance with the above XPS results and facts that such geometry is characteristic of known MoS_2_–organic compounds with other organic guests [11,30].

For modeling the organic layers, their thicknesses were set equal to those determined from the PXRD data for each compound. The initial conformation of MR molecules within these layers was taken as identical to the bent conformation, with the head group inclined to the long-chain alkyl tail, revealed previously by single-crystal XRD study [27] (Figure S2). Importantly, the DFT calculations have showed that this conformation of the MR cation, stabilized by intramolecular interactions, is a preferable one also in the water surrounding [27].

**Figure 5:**
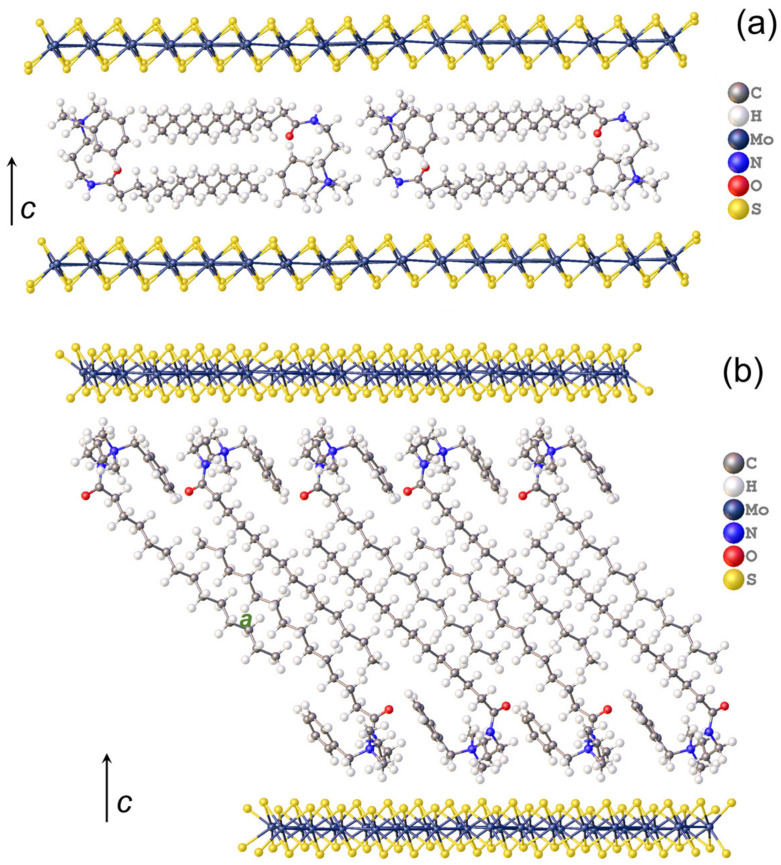
Model of LC1 (**a**) and LC2 (**b**) viewed perpendicularly to the c-axis.

**Figure 6:**
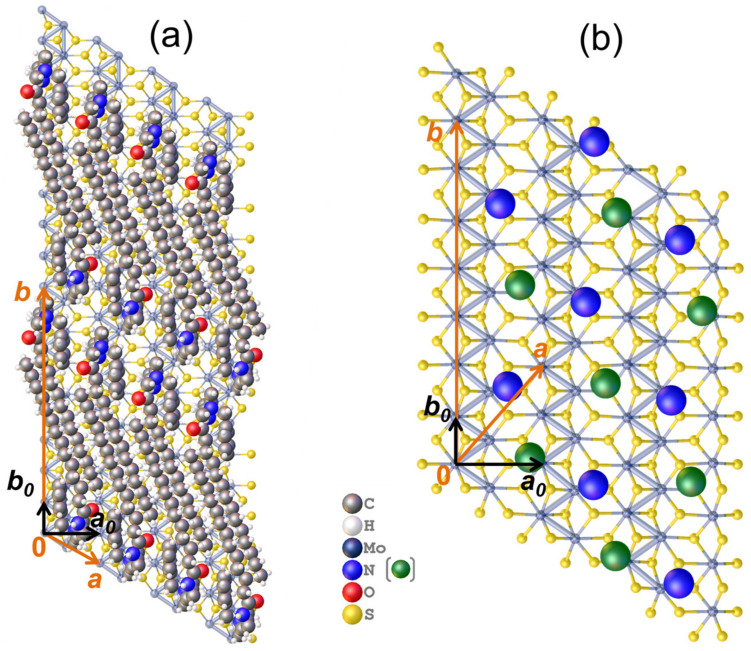
Disposition of MR molecules on MoS_2_ layer in LC1 (**a**) and LC2 (**b**) with reflection of the cell axis relation between the LCs (*a*, *b*) and MoS_2_ sheet (*a*_0_, *b*_0_). For clarity, only the quaternary nitrogen atoms colored differently for the oppositely directed molecules are shown in (**b**).

For modeling the structure of LC1, having the smallest interlayer distance, the dimer fragment of the MR chloride crystal packing containing two closely spaced oppositely directed molecules was used as a building block to populate the interlayer space by the bilayer-arranged MR cations. The satisfactory agreement with the organic layer thickness of LC1 is reached with the alkyl tails directed nearly parallel to the sulfide sheets and the planes of C-C zigzags roughly perpendicular to them (Figure 5a). The corresponding disposition of the MR molecules on the MoS_2_ layer, which allows the unreasonably short intermolecular contacts to be avoided, is described by the supercell, with $\bar{a}$ = $\bar{a_{0}}$ + $\bar{b_{0}}$, $\bar{b}$ = 8$\bar{b_{0}}$ (where $\bar{a_{0}}$ (5.65 Å) and $\bar{b_{0}}$ (3.23 Å) are the lattice parameters of MoS_2_ sheet) (Figure 6a). The composition of the resultant hybrid structure (MR)_x_MoS_2_ with x = 1/8 is in good agreement with the experimental elemental composition of LC1 (x = 0.14).

In the model of LC2, characterized by the greatest *c*-value, the thickness of the organic interlayer (25 Å) is best reproduced with the MR molecules arranged in a layered structure with coplanar disposition of the ammonium cationic heads adjoining to the sulfide sheets and the alkyl chains inclined to the heads plane (Figure 5b). In fact, this packing motif of the MR layer is identical to that in the above-mentioned MR crystalline salt (Figure S2). In order to inscribe such an MR layer in the periodic hetero-layered model, the commensurate relation between the in-layer periodicities of the MoS_2_ sheet and the MR layer was chosen as schematized in Figure S3. The packing density of such MR layer is slightly reduced as compared to the crystal (by approximately 8%) in order to reproduce the experimental MR/MoS_2_ molar ratio of LC2 (0.27) with reasonable accuracy. The periodic starting model of LC2 thus had the in-layer dimensions of $\bar{a}$ = 2$\bar{a_{0}}$ + 3$\bar{b_{0}}$, $\bar{b}$ = 7$\bar{b_{0}}$ and the composition (MR)_2/7_MoS_2_ (Figure 6b). Both models were then optimized by periodic DFT calculations using the plane wave (PW) basis set and Perdew–Burke–Ernzerhof (PBE) functional.

### 2.4. Optimized Structures of LCs

**Figure 7:**
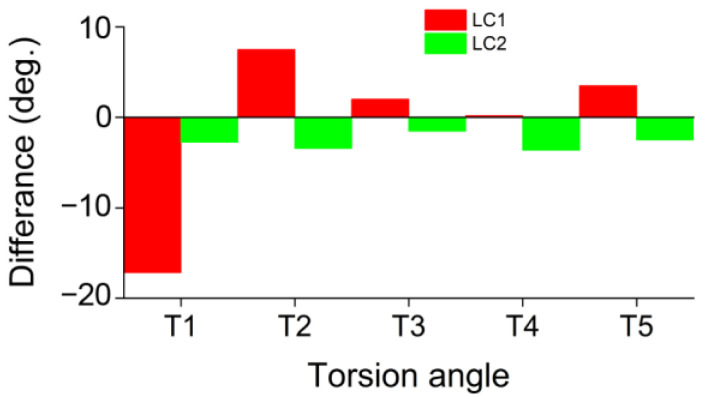
Difference between the MR torsion angles T1–T5 in LCs and crystal salt.

The selected structural parameters of the optimized LCs structures are presented in Table S4, supplemented by Figure S4. After optimization, the conformers of MR cation of both LCs remain generally similar to the starting one. However, some distortions of the head configuration compared to the crystal structure of MR occur. They are more pronounced in the case of LC1, as evidenced by the torsion angles T1–T5 values (Table S5) and structures overlay in Figure S5. The difference between the torsion angles in LCs and crystalline MR is illustrated by the histogram in Figure 7. The noticeable changes of T1 and T2 values observed for LC1, reflect the turn of carbonyl group towards the aromatic ring (T1) and approaching of this ring to alkyl chain and amide group (T2) (Figure S5).

**Figure 8:**
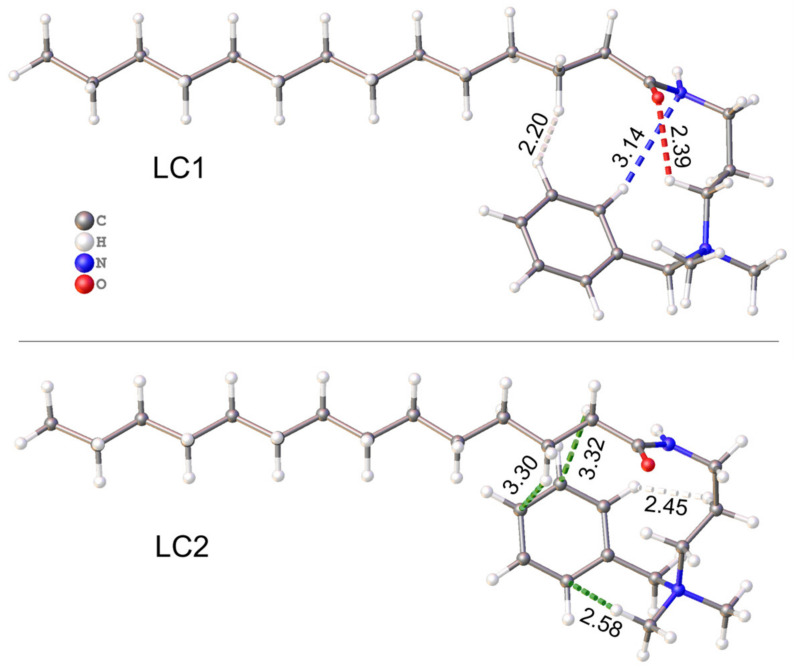
Intramolecular bonding in MR molecules of LCs. The legend for LC2 is the same as for LC1.

Similar to the crystal state, the folded configuration of MR is evidently favored in the hybrid structures by interactions intramolecular. In case of LC1, the strongest intramolecular bonding is provided by the CH…O (2.39 Å) and CH…N (3.14 Å) hydrogen bonds and hydrophobic H…H contact of the phenyl and alkyl hydrogen atoms (2.20 Å) (Figure 8). In LC2, the intramolecular bonding realizes due to the CH…π contacts of methyl and methylene substituents (Figure 8). The closest H…C_Ph_ contact of CH_3_ group (2.58 Å) is even shorter by 0.15 Å than the corresponding contact in the crystalline MR structure. The weaker CH…π contacts with ~3.3 Å distance involving the H atoms of the tail were identified as the bonding ones by QTAIM analysis described below. In addition, the phenyl ring is bound by the H…H contact with methylene groups of amidopropyl linker (2.45 Å), as can be seen in Figure 8.

In both LCs, the intermolecular bonding within the organic layer is caused by several CH…O and CH…C_π_ contacts (with the shortest distances amounting to, respectively, 2.34 Å and 2.46 Å for LC1 and 2.62 Å, 2.65 Å for LC2) and by numerous hydrophobic H…H interactions of aliphatic fragments. Notice that similar types of intermolecular contacts have been identified in the crystal packing of pure MR [27].

**Figure 9:**
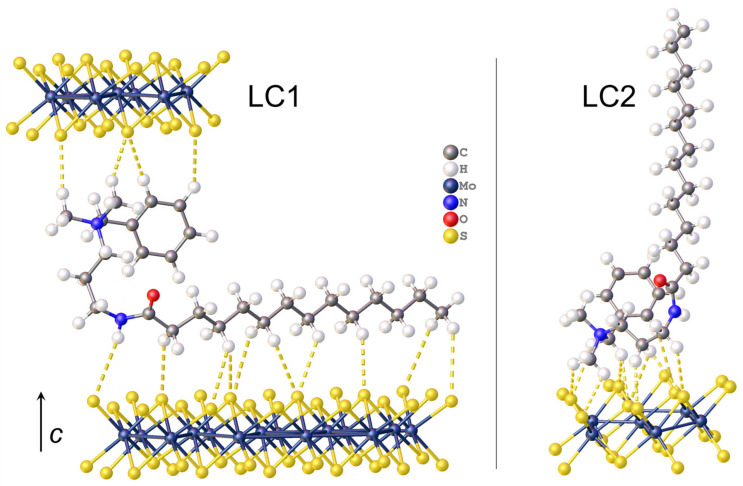
Sulfur surrounding of MR molecule in LCs with indication of bonding H…S contacts. The legend for LC2 is the same as for LC1.

The host–guest interactions in LCs are based exclusively on CH…S bonding contacts. In case of LC1, both the head and tail fragments can participate in such bonding (Figure 9). The shortest CH…S contacts of LC1, ranging in distance from 2.60 to 2.86 Å are established by the methyl and phenyl hydrogen atoms. The more numerous contacts of alkyl tail are somewhat longer (2.96–3.51 Å). However, due to a high slope angle of C–C zigzag plan to sulfide sheet (78°) in LC1, only one-half of alkyl hydrogen atoms of the tail can interact with sulfur. Unlike LC1, the spatial geometry of LC2 implies the MR-MoS_2_ contacts only for the H atoms neighboring to the quaternary nitrogen as illustrated by Figure 9. Among them, the methyl and methylene groups provide the shortest contacts with the distance 2.43–3.25 Å.

### 2.5. Quantification of Bonding Interactions in LCs

QTAIM analysis of the electron density function ρ(r) calculated for the optimized hybrid structures of LCs has revealed a sets of (3,−1) bond critical points (BCPs) on the binding pathways. These BCPs are characteristic of the intermolecular noncovalent interactions appearing in these systems. The energies of these interactions were estimated using the semi-empirical correlation between the bonding energy and the electronic potential energy density value [V(r)] at the BCP found early by Espinosa, Mollins and Lecomte [33]. The detailed characteristics of noncovalent interactions are given in Tables S6 and S7 for LC1 and LC2, respectively. The summary energetic effects of interactions are presented in Table 1.

The calculation results show that the cumulative effect of the intramolecular interaction of the MR molecule is higher in the case of LC1, in which these molecules, confined in a smaller interlayer space, adopt a more distorted conformation as compared to the crystal state. As to the in-layer intermolecular interaction of MR, it is provided in both hybrid compounds by a series of H…H hydrophobic interactions between the aliphatic fragments of neighboring molecules. These interactions account for 29% and 23% of the total interaction energy of LC1 and LC2, respectively. The dominant role of hydrophobic interactions in the MR intermolecular bonding that typifies the MR crystals [27] is thus preserved in the LCs despite of somewhat lesser density of MR packing (LC1) and reduced number of nearby molecules (LC2) in the interlayer as compared to the crystal packing. It is worth noting that hydrophobic intermolecular interaction is often of great significance for binding of surfactant molecules among themselves and with other organic species including peptides and DNA [34,35,36]. The contribution of other intermolecular bonds, the CH…O, CH… C_π_ ones, is lower, though also significant, for stabilization of the MR layer.

The strength of the MR-to-MoS_2_ bonding is thought to be a key factor influencing a self-assembly process of corresponding hetero-structures. As discussed above, in both obtained LCs, this bonding realizes a trough of individually weak CH…S contacts. Importantly, their cumulative effect provides the largest contributions to the total energy of interactions of each hybrid system (33% in LC1 and 53% in LC2). These effects explain a moving force of the assembly reaction and stability of the obtained LCs. Interestingly, the energy values of the cation–MoS_2_ interaction calculated for both systems are comparable in value, reaching −14.4 kcal/mol and −12.7 kcal/mol in LC1 and LC2, respectively.

## 3. Materials and Methods

### 3.1. Preparation of Layered Compounds

Initially, the crystalline LiMoS_2_ was prepared by treating purified natural molybdenum disulfide (DM-1, Scopin Factory, Scopin, Russia) with a particle size (95%) smaller than 7 μm with an excess of 1.6 M n-butyllithium solution in hexane (Aldrich) for 1 week, washing in hexane and drying in vacuum. The obtained compound was immersed in bi-distilled water, sonicated for 15 min, and stirred on a magnetic stirrer for 30 min to prepare the 1 mg·mL^−1^ dispersion of LiMoS_2_. The powdered MR-MoS_2_ layered compounds have been obtained by mixing aqueous dispersions of MoS_2_ with water solutions of benzyldimethyl{3-[(1-oxotetradecyl)amino]propyl}ammonium chloride (Infamed, Vidnoye, Russia) containing 0.14 (LC1) or 0.5 (LC2) mole of the salt per 1 mole of MoS_2_. After stirring of the reaction mixture on a magnetic stirrer for 2 h, the precipitate formed during the stirring, was collected by centrifugation, washed by water, and dried in vacuum. The composition of the products was determined from elemental analysis data (C, H, N, Mo). Found (calculated) for (MR)_0.14_MoS_2_ (LC1): C 20.48 (20.17), N 1.87 (1.81), H 2.81 (3.04), Mo 43.85 (44.3); for (MR)_0.27_MoS_2_ (LC2): C 31.33 (31,31), N 2.70 (2.81), H 4.14 (4.72), Mo 36.1 (35.7).

### 3.2. Material Characterization

Powder X-ray diffraction (PXRD) phase analysis was performed with a D8 Advance (Bruker AXS, Karlsruhe, Germany) diffractometer in the Bragg–Brentano focusing geometry using CuK_α_ radiation, the angular step was 0.02°, and the scan rate was 0.5–2 deg min^−1^. The samples were placed on flat holders. Powder patterns were processed with DIFFRACplus (Bruker AXS) software.

X-ray photoelectron spectra were acquired with an Axis Ultra DLD (Kratos, Stretford, UK) spectrometer using monochromatized Al Kα (1486.6 eV) radiation at an operating power of 150 W of the X-ray tube. Survey and high-resolution spectra of appropriate core levels were recorded at pass energies of 160 and 40 eV and with step sizes of 1 and 0.1 eV, respectively. Sample area of 300 μm × 700 μm contributed to the spectra. The samples were mounted on a sample holder with two-sided adhesive tape, and the spectra were collected at room temperature. The base pressure in the analytical UHV chamber of the spectrometer during measurements did not exceed 10^−8^ Torr. The energy scale of the spectrometer was calibrated to provide the following values for reference samples (i.e., metal surfaces freshly cleaned by ion bombardment): Au 4f_7/2_—83.96 eV, Cu 2p_3/2_—932.62 eV, Ag 3d_5/2_—368.21 eV. The spectra were charge-corrected to give the C 1s component attributed to C–C/C–H aliphatic bonds a binding energy of 285.0 eV. After charge referencing, a Shirley-type background with inelastic losses was subtracted from the high-resolution spectra. The Mo 3d spectra of LCs were fitted with 3d_3/2_–3d_5/2_ spin-orbit doublets with splitting of ~3.2 eV and branching ratio of 3/2 and the S 2p spectra were fitted with S 2p_3/2_–S 2p_1/2_ doublets with spin-orbit splitting of ~1.2 eV and branching ratio of 2.

The CHN content in the samples was determined with a Carlo Erba Elemental Analyzer 1106 (C, H, N) and the Mo content with a VRA 30 Carl Zeiss X-ray fluorescent spectrometer.

### 3.3. Quantum-Chemical Calculations

DFT (PW-PBE-D) periodic calculations were performed in VASP package [37,38,39,40] Projector augmented wave (PAW) pseudopotentials [41,42] were used for all atoms to describe core electrons. The contribution of valence electrons was described as a series of plane waves with kinetic energy cutoff 545 eV. Exchange and correlation terms of total energy were described by a PBE functional [43] with Grimme D3 dispersion correction [44]. Electron density function for topological analysis was obtained in separate single point calculations with kinetic energy cutoff 800 eV. Topological analysis was carried out by using the AIM program, a part of ABINIT software [45].

## 4. Conclusions

From our study, it emerged that the molecules of quaternary ammonium amphiphile antiseptic miramistin tend to self-organize with monolayer MoS_2_ sheets, dispersed in water solution, into nanoscale ordered layered hybrids. There are two stable hybrid compounds identified in this system, which differed in the content of miramistin by two times. Both compounds are characterized by regular alternation of the organic (miramistin) and inorganic (MoS_2_) monolayers; however, the thicknesses of organic layers differs significantly between the two structures, that evinces the differences in the packing of their organic layers. The results of structural modeling of both hybrid structures are consistent with the bent conformation of their miramistin molecules sandwiched between the layers of MoS_2_. As with pure crystalline miramistin, this conformation is stabilized in the hybrids by intramolecular bonding interactions; they are more important for the molecules confined in the interlayer space of smaller thickness. The hydrophobic H…H bonding inherent to alkylammonium surfactants was found to prevail over the in-layer intermolecular interactions of miramistin molecules occurring in 2D interlayer spacing of MoS_2_. The host-guest miramistin–MoS_2_ interaction is realized by means of a series of CH…S bonding contacts, which are individually weak but numerous, so their cumulative effect produces the largest contributions to structure stabilization of both obtained hybrids. This work promotes general understanding of the organic molecules hybridization with sulfide surfaces. The obtained results are hoped to be used in future development of novel MoS_2_-based photothermal agents.

## Figures and Tables

**Table 1:** Summary of bonding interaction energies of LCs (kcal/mol per mole of MR).

Interaction	LC1	LC2
Intramolecular	−7.5	−3.0
Intermolecular:		
H…H	−12.7	−12.7
CH…O	−9.0	−2.9
Host-guest	−14.4	−12.7
Total	−43.6	−24.1

## Data Availability

The data presented in this study are available in the article.

## Supplementary Materials

The following supporting information can be downloaded at: https://www.mdpi.com/article/10.3390/molecules28041702/s1, Figure S1: C1s (a), N 1s (b), Mo 3d (c) and S 2p (d) photoelectron spectra of LC2; Table S1: Parameters of components in C 1s and N 1s photoelectron spectra of LCs; Table S2: Parameters of components in Mo 3d photoelectron spectra of LCs; Table S3: Parameters of components in S 2p photoelectron spectra of LCs; Figure S2: Crystal packing of Miramistin molecules in the chloride salt viewed along a-axis; Figure S3: Superimposition of the MR chloride lattice on MoS_2_ network, adjusted commensurate MR cell and corresponding periodicity of MR layer in LC2 model; Table S4: Selected structural parameters of LCs; Figure S4: Notations for selected structural parameters of LCs; Table S5: Selected torsion angles of MR molecule (deg.) in LCs and crystalline salt. Figure S5: Superimposed structures of MR in LC1 and crystalline salt; Table S6: Characteristics of noncovalent interactions in LC1; Table S7: Characteristics of noncovalent interactions in LC2; Calculated models, LC1 and LC2 res files.
