# Supplementary material for: Structural Significance of Hydrophobic and Hydrogen Bonding Interaction for Nanoscale Hybridization of Antiseptic Miramistin Molecules with Molybdenum Disulfide Monolayers
Source: Molecules. 2023 Feb 10;28(4):1702. doi: 10.3390/molecules28041702 (PMC9966762; doi:10.3390/molecules28041702)
Supplement: Supplementary file 1 [file molecules-28-01702-s001.zip › molecules-2205047-supplementary.pdf]

Supplementary Materials

Structural Significance of Hydrophobic and Hydrogen Bonding Interaction for Nanoscale Hybridization of Antiseptic Miramistin Molecules with Molybdenum Disulfide Monolayers

Alexander S. Goloveshkin,[a] Natalia D. Lenenko,[a] Alexander V. Naumkin,[a] Alexandre S. Golub *[a]

[a] A.N. Nesmeyanov Institute of Organoelement Compounds, Russian Academy of Sciences, Vavilova St. 28, Moscow, 119934, Russia

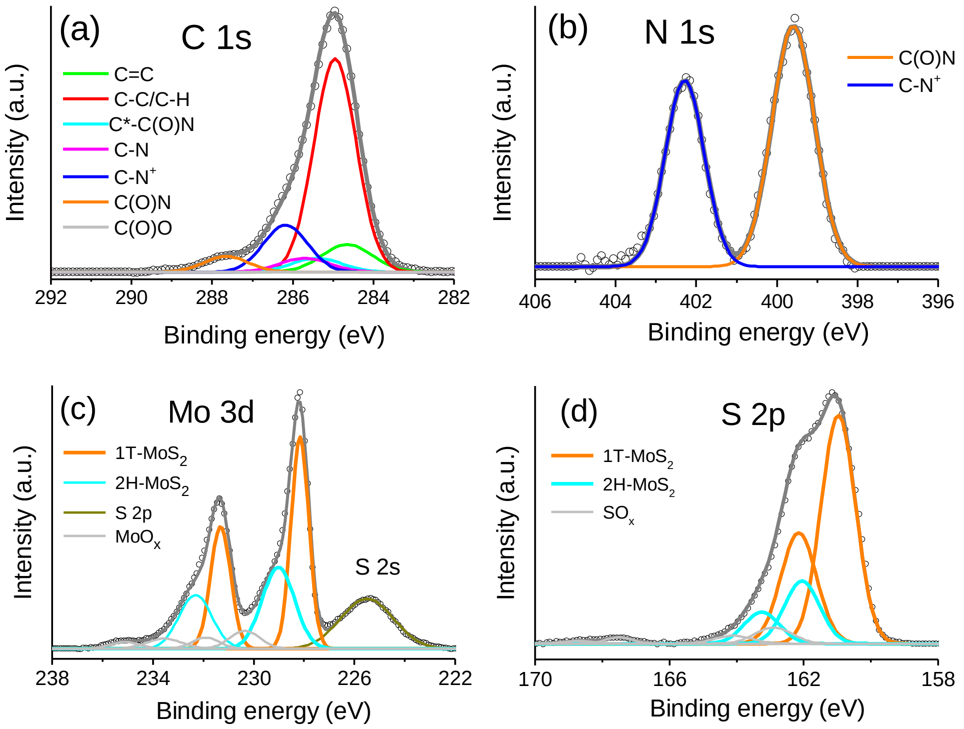

Figure S1. C1s (a), N 1s (b), Mo 3d (c) and S 2p (d) photoelectron spectra of LC2.

Table S1. Parameters of components in C 1s and N 1s photoelectron spectra of LCs (E_b is a binding energy, W is a peak width, I_{rel} is a relative intensity).

Sample		C 1s							N 1s	
		C=C	C-C/C-H	C*-C(O)N	C-N	C-N$^+$	C(O)N	C(O)O	C-N$^+$	C(O)N
LC1	E_b, eV	284.7	285.0	285.4	285.7	286.3	287.5	289.2	402.3	399.6
	W, eV	1.25	1.23	1.25	1.25	1.2	1.2	1.25	0.97	1.02
	I_{rel}	0.23	0.49	0.04	0.04	0.15	0.04	0.02	0.47	0.53
LC2	E_b, eV	284.7	285.0	285.4	285.7	286.2	287.7		402.3	399.6
	W, eV	1.25	1.05	1.25	1.25	1.1	1.1		0.97	1.06
	I_{rel}	0.10	0.62	0.05	0.05	0.14	0.05		0.42	0.58

Table S2. Parameters of components in Mo 3d photoelectron spectra of LCs (E_b is a binding energy, W is a peak width, I_{rel} is a relative intensity).

		Mo1 (1T)		Mo2 (2H)		Mo3		Mo4	
		$3d_{5/2}$	$3d_{3/2}$	$3d_{5/2}$	$3d_{3/2}$	$3d_{5/2}$	$3d_{3/2}$	$3d_{5/2}$	$3d_{3/2}$
LC1	E_b, eV	228.3	231.5	229.1	232.4	230.4	233.6	232.3	235.5
	W, eV	0.7	0.82	1.22	1.3	1.1	1.2	1.2	1.4
	I_{rel}	0.52		0.34		0.07		0.07	
LC2	E_b, eV	228.2	231.3	229.0	232.2	230.4	233.6	231.9	235.1
	W, eV	0.71	0.80	1.1	1.3	1.2	1.4	1.1	1.4
	I_{rel}	0.52		0.36		0.07		0.04	

Table S3. Parameters of components in S 2p photoelectron spectra of LCs (E_b is a binding energy, W is a peak width, I_{rel} is a relative intensity).

		S1 (1T)		S2 (2H)		S3		S4	
		S $2p_{3/2}$	S $2p_{1/2}$	S $2p_{1/2}$	S $2p_{3/2}$	S $2p_{3/2}$	S $2p_{1/2}$	S $2p_{3/2}$	S $2p_{1/2}$
LC1	E_b, eV	161.1	162.3	162.2	163.4	163.3	164.5	167.7	168.9
	W, eV	1.03	1.05	1.05	1.05	1.1	1.1	0.8	0.9
	I_{rel}	0.72		0.20		0.06		0.02	
LC2	E_b, eV	161.0	162.2	162.1	163.3	162.9	164.1	167.5	168.7
	W, eV	1.03	1.06	1.05	1.05	1.1	1.1	0.8	0.9
	I_{rel}	0.73		0.20		0.05		0.02	

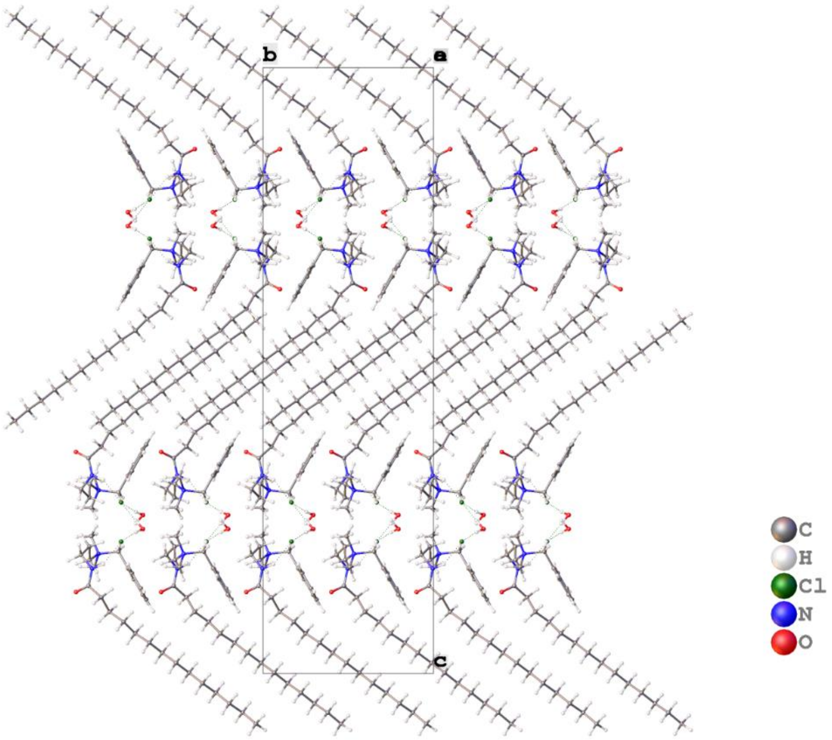

Figure S2. Crystal packing of Miramistin molecules in the chloride salt viewed along *a*-axis according to [Dolgushin, F.M., et all. *Acta Crystallogr C Struct Chem* 2019, *75*, 402–411].

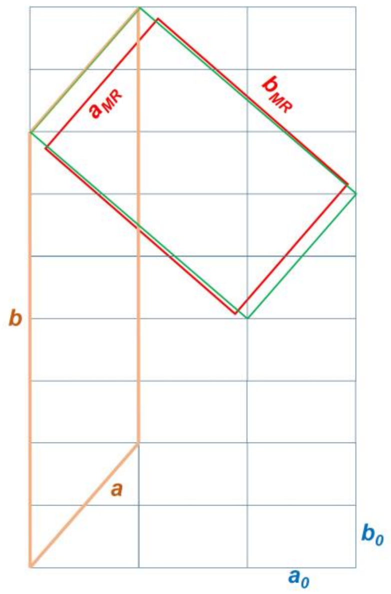

Figure S3. Superimposition of the MR chloride lattice (red) on MoS_2 network (blue), adjusted commensurate MR cell (green) and corresponding periodicity of MR layer in LC2 model. The cell dimensions are as follows: red, a_{MR} = 8.92 Å, b_{MR} = 13.07 Å; blue, a_0 = 5.65 Å, b_0 = 3.23 Å; green $\bar{a} = \bar{a}_0 + 2\,\bar{b}_0$, $\bar{b} = 2\,\bar{a}_0 - 3\bar{b}_0$; orange, $\bar{a} = \bar{a}_0 + 2\,\bar{b}_0$ (8.582 Å), $\bar{b} = 7\,\bar{b}_0$ (22.61 Å).

Table S4. Selected structural parameters of LCs.

Parameter	LC1	LC2
Space group	P1	P1
Cell content	$(MR)_2(MoS_2)_{16}$	$(MR)_4(MoS_2)_{14}$
a (Å)	6.60	8.58
b (Å)	25.87	22.61
c (Å)	16.0	31.0
α (°)	90	90
β (°)	90	90
γ (°)	119.32	41.17
V, A^3	2380.1	3960.0
ρ, g/cm^3	2.35	1.617
Mo-Mo (Å)	2.78-2.83	2.73-2.85
Mo-Mo-Mo (deg)	69.6-6-73.0	69.0-76.9
Mo-S (Å)	2.38-2.55	2.37-2.55
S-S Δz1,Δz2 (Å)	3.44, 2.62	3.44, 2.64
Mo-Mo Δz (Å)	0.13	0.13
N-S Δz1, Δz2 (Å)	4.45, 4.04	4.04, 3.64
N-N Δz (Å)	4.47	20.27
Tails inclination to sulfide layers (˚)	1.4	44-47

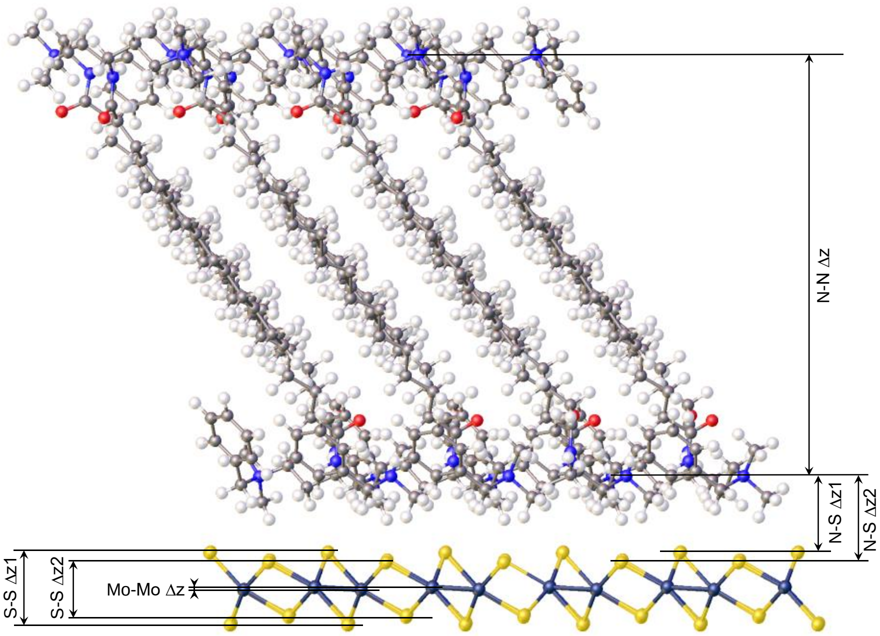

Figure S4. Notations for selected structural parameters of LCs.

Table S5. Selected torsion angles of MR molecule (deg.) in LCs and crystalline salt.

Angle *	Crystal	LC1	LC2
T1	55.13	37.95	52.39
T2	67.85	75.32	64.41
T3	176.97	178.98	175.44
T4	70.18	70.35	66.56
T5	87.92	91.40	85.42

* Notations correspond to Figure 1 of the main text.

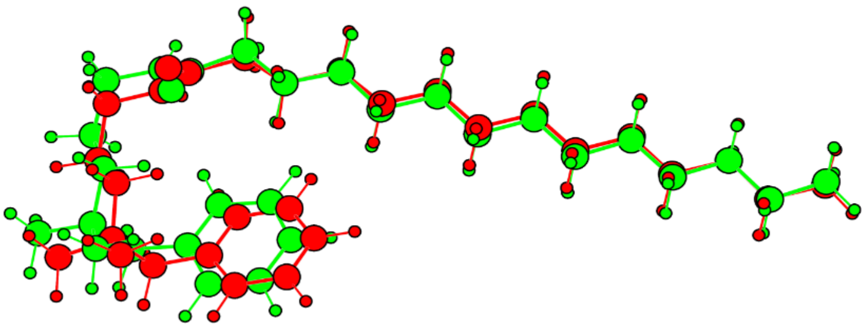

Figure S5. Superimposed structures of MR in LC1 (green) and crystalline salt (red).

Table S6. Characteristics of noncovalent interactions in LC1.

Interaction	Number of contacts	DH…A, H…H (Å)	E (kcal/mol)	∑E* (kcal/mol per cell)	∑E (kcal/mol per mole of cation)
		Intramolecular			
CH…O_{CO}	2	2.31 to 2.39	2.5 to 2.9	-5.3	-2.65
CH…N	2	2.84 to 3.14	0.5 to 0.8	-1.3	-0.65
CH...H_{Ph}	7	2.20 to 2.69	0.4 to 1.7	-6.5	-3.25
H...H	1	2.16	1.8	-1.8	-0.90
		Intermolecular			
C-H…O_{CO}	5	2.34 to 3.44	0.2 to 2.4	-4.8	-2.4
C-H…C_{π}	8	2.46 to 3.78	1.0 to 2.4	-10.6	-5.3
H…H	47	2.16 to 2.94	0.2 to 1.1		-12.7
		Host-guest			
CH…S (tail)	24	2.81 to 3.67	0.2 to 1.3	-13.7	-6.85
CH…S (CH_3)**	7	2.60 to 3.35	0.4 to 2.2	-8.1	-4.05
CH…S (CH_2)**	1	3.09	0.7	-0.7	-0.35
CH…S (Ph) Total	4	2.66 to 3.26	0.4 to 1.8	-6.3	-3.15
					-43.6

* Cell composition is $(MR)_2(MoS_2)_{16}$.
** CH_3 and CH_2 denote corresponding groups of the head.

Table S7. Characteristics of noncovalent interactions in LC2.

Interaction	Number of contacts	DH…A, H…H (Å)	E (kcal/mol)	∑E* (kcal/mol per cell)	∑E, (kcal/mol per cation)
		Intramolecular			
CH...H_{Ph}	5	2.35 to 2.63	0.5 to 0.9	-3.6	-0.9
CH…C_{π}	10	2.58 to 3.32	0.3 to 2.1	-8.5	-2.1
		Intermolecular			
CH…O_{CO}	8	2.62 to 3.15	0.3 to 1.3	-5.2	-1.3
CH…C_{π}	9	2.65 to 3.05	0.4 to 1.3	-6.5	-1.6
H...H	54	2.39 to 2.92	0.2 to 1.3	-21.9	-5.5
		Host-guest			
CH…S (CH_3)**	11	2.43 to 3.01	1.0 to 3.8	-24.4	-6.1
CH…S (CH_2)**	25	2.56 to 3.25	0.5 to 2.5	-25.4	-6.4
CH…S (Ph)	2	3.28 to 3.50	0.3 to 0.6	-0.9	-0.2
Total					-24.1

* Cell composition is $(MR)_4(MoS_2)_{14}$.
** CH_3 and CH_2 denote corresponding groups of the head.
